# *In silico* multi-epitope-based vaccine design for *Mycobacterium avium* complex species

**DOI:** 10.3389/fimmu.2025.1589083

**Published:** 2025-06-05

**Authors:** Leah Kashiri, Wonderful T. Choga, Tinashe Musasa, Pasipanodya Nziramasanga, Rutendo B. Gutsire, Lynn S. Zijenah, Norman L. Mukarati, Simani Gaseitsiwe, Sikhulile Moyo, Nyasha Chin’ombe

**Affiliations:** ^1^ Medical Microbiology Unit, Department of Laboratory Diagnostic and Investigative Sciences, Faculty of Medicine and Health Sciences, University of Zimbabwe, Harare, Zimbabwe; ^2^ Department of Medical Sciences, Botswana Harvard Health Partnership, Gaborone, Botswana; ^3^ School of Allied Health Sciences, Faculty of Health Sciences, Gaborone, Botswana; ^4^ Department of Applied Biology and Biochemistry, National University of Science and Technology, Bulawayo, Zimbabwe; ^5^ Immunology Unit, Department of Laboratory Diagnostic and Investigative Sciences, Faculty of Medicine and Health Sciences, University of Zimbabwe, Harare, Zimbabwe; ^6^ Department of Clinical Veterinary Sciences, Faculty of Veterinary Sciences, University of Zimbabwe, Harare, Zimbabwe; ^7^ Department of Immunology and Infectious Diseases, Harvard T.H. Chan School of Public Health, Boston, MA, United States; ^8^ School of Health Systems and Public Health, University of Pretoria, Pretoria, South Africa; ^9^ Division of Medical Virology, Faculty of Medicine and Health Sciences, Stellenbosch University, Tygerberg, South Africa

**Keywords:** Epitopes, Mycobacterium avium complex, Vaccine, Antigen85, mycolyltransferase, Th1 helper T-cell, immunodominance, promiscuous epitopes

## Abstract

**Introduction:**

The *Mycobacterium avium* complex (MAC)—comprising *M. colombiense*, *M. avium*, and*M. intracellulare*—is an emerging group of opportunistic pathogens responsible for significant morbidity and mortality, particularly in immunocompromised individuals. Despite this growing burden, no vaccines currently provide cross-species protection. In silico vaccine design offers a rapid, cost-effective strategy to identify immunogenic epitopes and assemble multi-epitope constructs with optimized safety and efficacy. Accordingly, we aimed to develop a candidate multi-epitope vaccine (MEV) targeting conserved antigens across multiple MAC species.

**Methods:**

From a genomic survey of nontuberculous mycobacteria (NTM) in Zimbabwe, we assembled complete genomes for *M. colombiense* (MCOL), *M. avium* (MAV), and *M. intracellulare* (MINT). Using both local and global reference datasets, we screened the conserved immunodominant proteins 85A, 85B, and 85C for high-affinity T-helper lymphocyte (THL) epitopes. Promising epitopes were further evaluated for antigenicity, immunogenicity, physicochemical stability, and population coverage.

**Results:**

Epitope mapping across the nine target proteins yielded 82 THL epitopes predicted to bind 13 MHC class II (DRB*) alleles, ensuring broad coverage within Zimbabwean and pan-African populations. Clustering analyses consolidated 26 unique epitopes into 11 consensus peptides, 65.4% of which derived from the 85B proteins. *In silico* immune simulations predicted robust humoral and cellular responses, including elevated IgG titers, T-helper and T-cytotoxic cell proliferation and increased secretion of IFN-γ and IL-2 following MEV administration.

**Conclusion:**

These findings indicate that our construct possesses strong immunogenic potential and cross-species applicability. We present here a rationally designed MEV candidate that merits further experimental validation as a broad-spectrum vaccine against multiple MAC species.

## 1 Introduction

*Mycobacterium avium* complex (MAC) encompasses a group of twelve species of mycobacteria that are opportunistic pathogens responsible for significant morbidity and mortality in both humans and animals (1, 2). To improve on clarity, may we request change of this statement to 'MAC species cause pulmonary disease in humans and animals. They are clinically significant both in immunocompromised patients—such as people living with HIV/AIDS or those with chronic lung disease—and, less commonly, in otherwise healthy individuals. (3). In animals, MAC infections pose significant threats to livestock and wildlife resulting in economic losses and serving as potential reservoirs for zoonotic transmission (4, 5). Infections due to MAC species are increasing globally, particularly among immunocompromised individuals and patients with underlying lung disease, with notable prevalence reported in Australia, America, Europe, and Asia, thereby underscoring the urgent need for effective vaccine strategies worldwide (1, 3, 6–9). The growing global burden of MAC infections and multidrug resistance among MAC calls for an urgent and constant ‘*One Health Approach*’ in development of effective prevention and control strategies in both humans and animals (2). This vaccine development effort aligns with the ‘*One Health approach*’ addressing human, animal and environmental health by targeting pathogens at the human-animal-environment interface. Despite the widespread use of Bacillus Calmette–Guérin (BCG) as a vaccine for tuberculosis and also providing partial immunity to NTM infections, further studies to either increase the efficacy of the BCG/recombinant BCG vaccine or to create new vaccines or booster vaccines that induce an optimal immune response against NTM is required (10). To address this waning efficacy, booster vaccines are essential as they help achieve long-term immunity (11). However, the recent advances in vaccine technology, *in silico* predictions provide a more efficient, cost-effective alternative for screening candidate epitopes that can elicit strong immune responses, identifying and optimizing vaccine candidates used in the development of therapeutics and vaccines.

Given that effective vaccines must enhance immune mechanisms responsible for pathogen elimination, understanding the nature of pathogen clearance becomes essential. Pathogen clearance often relies on multi-specific, polyclonal, and robust T cell-mediated responses. Major histocompatibility complexes (MHCs), known as human leukocyte antigens (HLAs) in humans, are crucial for the host immune system, presenting antigenic peptides (epitopes) to CD8+ cytotoxic T cells (CTLs) and CD4+ T helper (Th) cells [Helper T-lymphocytes (HTL)] (12–14). HLA class I molecules present endogenous peptides to CTLs, while HLA class II molecules present exogenous peptides to HTLs (13). The HTLs have a key role in adaptive immunity. These activate the B-cells along with the CTLs for production of antibodies and eventually killing infected/damaged cells (15). The HTL epitopes for the selected protein can be calculated using the prediction tool for MHC-II epitope (http://tools.iedb.org/main/tcell/).

Epitope-based vaccines represent a novel approach for generating a specific immune response and avoiding responses against other unfavourable epitopes (like epitopes that may drive immunopathogenic or immune modulating responses) in the complete antigen. Potential advantages of epitope-based vaccines also include increased safety, the opportunity to rationally engineer the epitopes for increased potency and breadth, and the ability to focus immune responses on conserved epitopes (16, 17). The repertoire of peptides presented by HLAs is influenced by the structural features of the HLA binding groove and the peptide’s amino acid composition (18, 19). *In silico* tools can predict MHC-presented epitopes and profile immune escape mutations, though such analyses remain complex and underexplored for bacterial genomes. Additionally, pathogens frequently mutate within immunogenic epitopes to evade recognition by T cells and agents, posing significant challenges for developing potent vaccines and therapeutics for diseases like tuberculosis (TB) and others (20).

The antigen 85 (Ag85) complex, comprising a cascade of 85A, 85B, and 85C proteins is the main secretory antigen playing an important role in the pathogenicity of mycobacteria (21). Ag85 complex molecules are widely being explored as tools in diagnostic methods and in vaccine research including recombinant attenuated vaccines, DNA vaccines and subunit vaccines because of their ability to allow bacteria to evade host immune responses through preventing formation of phagolysosomes (21). These highly conserved fibronectin-binding proteins also promote immune responses in host by inducing the production of IFN-g and have been shown to confer protection against TB (22). Research into Ag85 proteins continues to hold great promise for improving TB vaccines, particularly in high-burden settings.

By leveraging computational approaches, it is possible to predict T-cell from MAC antigens, assess their immunogenicity, and design multi-epitope vaccine constructs. In this study, we utilized immunoinformatics to analyze the 85A, 85B, and 85C proteins of MAV, MINT and MCOL, the commonly found strains in Zimbabwe, to identify candidate epitopes for vaccine development. This work aims to contribute to the development of safe and effective vaccines to combat MAC infections in both humans and animals, addressing a critical need in the global fight against mycobacterial diseases.

## 2 Materials and methods

### 2.1 Screening for Ag85A-C genes of the three MAC species

From a genomic survey project using NTM samples in Zimbabwe, we generated complete MAV, MINT and MCOL genomes. These genomes provided a foundational dataset for vaccine modelling, enabling a genotyping approach through whole genome sequencing (WGS) to identify immunologically relevant targets. To enhance the multi-epitope vaccine design, we screened for Ag85A-C genes across the three MAC species. The amino acid sequences of the target proteins Ag85A, Ag85B, and Ag85C of MAV, MINT and MCOL were retrieved from the National Center for Biotechnology Information (NCBI) (https://www.ncbi.nlm.nih.gov) in FASTA format. Subsequently, the nine protein sequences were grouped into three multiple sequence alignments (MSAs), corresponding to Ag85A, Ag85B, and Ag85C for MAV, MINT, and MCOL. Each MSA was visualized using AliView (https://github.com/AliView/AliView), and conserved regions were identified from the alignments. The Epitope Conservancy Analysis tool, available through the Immune Epitope Database (IEDB; http://tools.iedb.org/conservancy/), was used to evaluate the variability of epitopes based on the sequence alignment of the three MAC species.

### 2.2 Prediction of T-cell and designing of the multi-epitope subunit vaccine

We used NetMHCIIpan 4.3 to predict MHC class II-binding peptides (NetMHCIIpan 4.3 - DTU Health Tech - Bioinformatic Services) for all the 3 MSA containing proteins (Ag85A, Ag85B, and Ag85C) for multiple MAC species. Epitope selection thresholds were based on established immunoinformatics criteria. Only epitopes that fulfilled multiple criteria, high antigenicity, strong MHC binding, and IFN-γ induction were shortlisted for vaccine construct design. These selection thresholds have been widely adopted in previous epitope-based vaccine design studies to ensure that the predicted peptides are likely to be immunogenic and broadly recognized across different HLA types (16, 17, 23). As a selection criteria, the strong binding promiscuous epitopes were considered for downstream analyses towards final vaccine construct. To enhance vaccine efficacy, the GPGPG linker was used to connect amino acid sequences, ensuring optimal individual functionality (23, 24). GPGPG linkers reduced junctional immunogenicity. Since immune adjuvants are a key requirement in vaccine formulation and play a critical role in enhancing the efficacy of vaccines, the *Mycobacterium tuberculosis* 50S ribosomal protein L7/L12 (RL7_MYCTU), P9WHE3 was retrieved from the UniProt database (https://www.uniprot.org/) and used as an adjuvant for the immune interaction based on its ability to act as an agonist for TLR437 (25). P9WHE3 was then integrated at the N-terminal of the construct and connected to the antigenic epitopes using the EAAAK linker to ensure improved expression, bioactivity stability and structural integrity (26, 27). The final vaccine construct was meticulously designed by assembling the adjuvant, epitopes, and linkers into a unified, functional structure to maintain the structural stability and immunological independence of the epitopes and adjuvant. To analyze epitope similarity with human surface proteins and minimize the risk of autoimmune reactions, BLASTp was also used. Epitopes with a similarity below 70% to human proteins are considered acceptable (28). The analysis was conducted using the BLASTp tool.

### 2.3 Determination of physicochemical characteristics, immunogenicity and allergenicity prediction

We evaluated the immunogenicity of the multiepitope subunits using the VaxiJen (VaxiJen v3.0) and the ANTIGENpro module of the SCRATCH protein predictor (Scratch Protein Predictor). Allergenicity was assessed with the AllerTOP v. 2.0 (29) and AlgPred servers (http://crdd.osdd.net/raghava/algpred/) to identify potential allergic reactions, ensuring the safety and efficacy of the predicted vaccine candidates. We used the ProtParam tool of the EXPASY database server (http://web.expasy.org/protparam/) to determine the physicochemical parameters (molecular weight, half-life, atomic composition, stability index and mean hydrophilicity) of the vaccine candidates’ antigens.

### 2.4 3D modelling of immunogenic polypeptides and protein subunits

For *ab initio* modelling, we utilized the Swiss-model (SWISS-MODEL), submitting the designed full length chimeric peptide sequence with default settings. One model (Swiss-Model ID: Q63Q02.1) encompassed the entire multi-epitope construct, while the second returned only a truncated peptide fragment and was therefore excluded from further consideration. Model Q63Q02.1 was subsequently validated using MolProbity metrics—MolProbity score, clashscore, and Ramachandran analysis (**Table 1**)—to ensure stereochemical quality before downstream analyses. Functional insights into the targets were derived by rethreading the models through the BioLiP protein function database (BioLiP). The resulting 3D models were visually inspected using PyMOL (PyMOL | pymol.org) for structural validation and analysis. To further evaluate the protein’s flexibility and dynamics, Normal Mode Analysis (NMA) was performed using the iMODS server (https://imods.iqf.csic.es/), which provided insights into residue coupling through the covariance matrix and defined the elastic network model to identify regions of rigidity and flexibility based on the stiffness of atomic interactions.

**Table 1:** Structural validation metrics for the chimeric MEV construct (Model Q63Q02.1).

Model ID	MolProbity Score	Clash score	Ramachandran Favored (%)	Ramachandran Outliers (%)
*Q63Q02.1*	1.73	3.64	88.35	1.94

### 2.5 Population coverage by HTL epitopes

Human leukocyte antigen (HLA) patterns differ across ethnic groups and geographical regions, making it essential to evaluate population coverage when designing effective vaccines. The IEDB population coverage tool (http://tools.iedb.org/population/) was used to calculate global human population coverage for the predicted HTL epitopes, ensuring their broad applicability. The 15-mer peptides overlapping by 14 amino acids were tested for binding to a set of 13 HLA class II alleles—HLA-DRB1*0101, DRB1*0301, DRB1*0302, DRB1*0401, DRB1*0701, DRB1*0802, DRB1*1101, DRB1*1102, DRB1*1301, DRB1*1302, DRB1*1501, and DRB5*0101—that have high population coverage in Zimbabwe and other African populations (http://www.allelefrequencies.net/hla6006a.asp?hla_population=2057).

## 3 Results

The amino acid sequences of nine proteins, 85A, 85B and 85C proteins of MAV, MINT and MCOL, were retrieved from the GenBank database to design a multi-epitope vaccine targeting MAC. The inclusion of Ag85 complex was guided by its high degree of conservation among mycobacterial species and its established immunogenicity as supported by its wide use in vaccine candidate development for TB (26, 30–32). Although nine proteins were selected, clustering and consensus analysis resulted in 11 distinct peptide sequences, reflecting inter-strain variability and epitope overlap. The eleven protein sequences were MSFIEKVRKLRGAAATMPR, MSFFEKLRGAAATMPRR, PRRLAIAAVGASLLSGVAVAAGGS, PRRLAIAAMGASLLSGL, RLAIAAVGASLLSGL, GLPVEYLEVPSPSMGRNI, SEKVRAWGRRLLVGAAAAVTLPGLIGIAGGAATAN, SEKVRAWGRRLLVGTAAAATLPG, AWGRRLVVGAAAAATLPGLIGLAGGAATAN, PGLPVEYLQVPSAGMGRNI and PVEYLQVPSAGMGRDIKVQFQS. SignalP 4.5 was used to assess functionality, revealing no signal peptides for proteins other than Ag85A/B/C. Functional protein sequences were then subjected to T-cell epitope prediction, identifying 17 high-affinity HTL epitopes for inclusion in the final vaccine construct. These epitopes overlapped with HTL epitopes (**Table 2**). A BLAST search against the UniProt database confirmed high conservation among the proteins, ranging from 27.7% to 100%.

**Table 2:** Characteristics of the primary structure of proposed multi-epitope vaccine candidate for MAC species calculated through ProtParam tool.

Characteristics of Vaccine	Assessment
*Number of amino acids*	423 aa
*Molecular weight (kDa)*	41.5 kDa
*Theoretical pI*	10.27
*Negatively charged residues (Asp + Glu)*	31
*Positively charged residues (Arg + Lys)*	50
*Extinction coefficient (M^-1^cm^-1^)*	20910
*Estimated half life*	30 hours (mammalian reticulocytes)
*Aliphatic index*	88.09
*Grand average of hydropathicity (GRAVY)*	0.099
*Instability index*	36.67 (Stable)
*Rama favoured score of tertiary structure*	88.35%
*z-score*	4.41
*Codon Optimization Index (CAI)*	0.80
*GC content (E coli as vector)*	64.38%
*Allergenicity (ALLERCATPRO + AlgPred)*	No evidence (non-allergenic)
*Antigenicity Score: Threshold (0.4)*	0.6120 (Probable ANTIGEN).

### 3.1 Construction of multi-epitope subunit vaccine.

**Figure 1:**
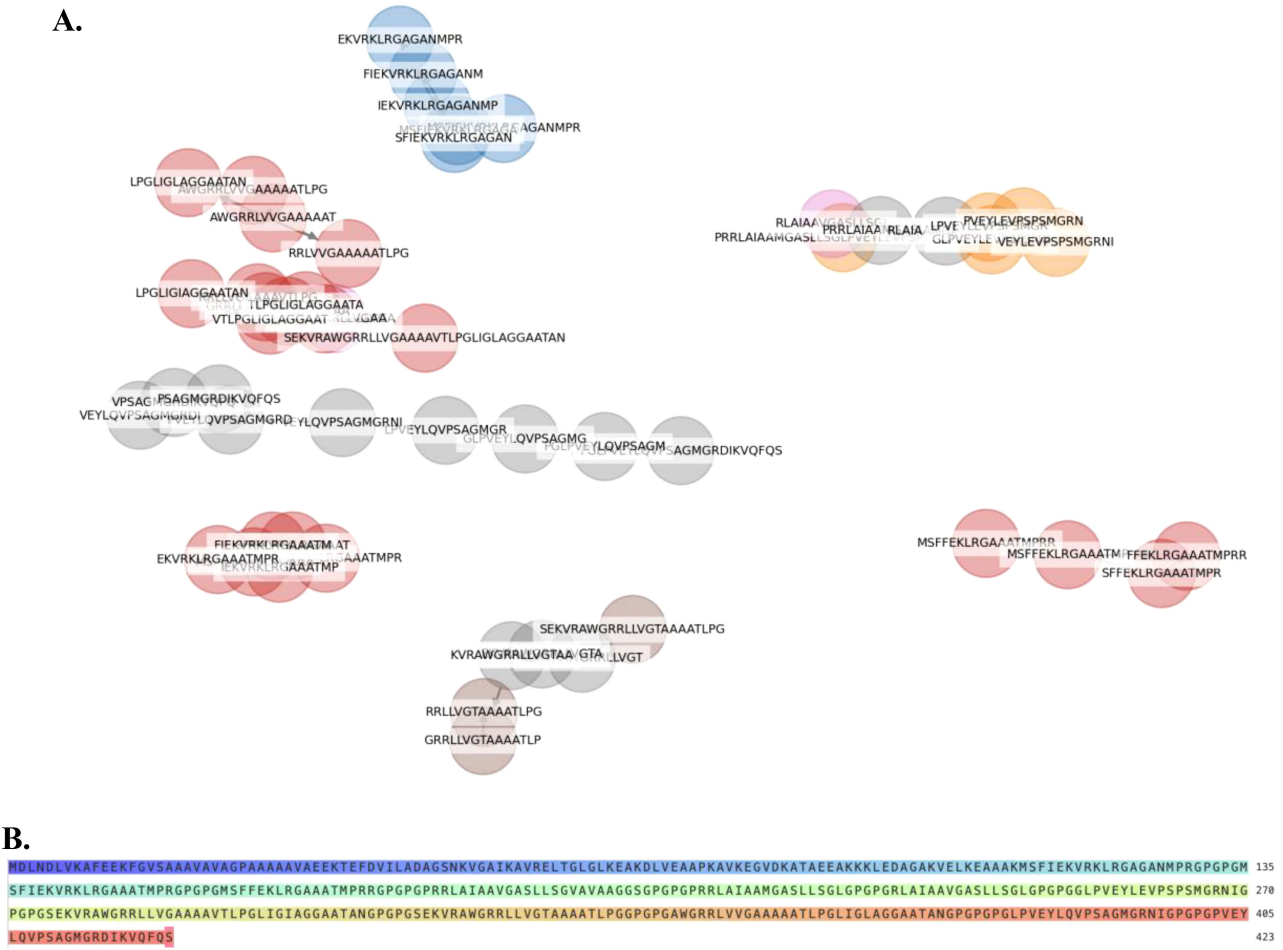
Helper T lymphocyte (HTL) Epitope Clustering and Multi-Epitope Vaccine Construct Design **(A)**. Predicted HTL 14-mer epitopes were grouped into clusters based on sequence similarity of their core binding regions. **(B)** A multi-epitope-vaccine construct was designed using consensus sequences derived from each epitope cluster.

A total of 7 clusters of overlapping 14-mers high-binding HTL epitopes were predicted (**Figure 1A**). Consequently, 7 consensus sequences representing each cluster were generated at 100% threshold. These were used in designing the chimera using GPGPG linkers. Additionally, an adjuvant was added to the amino terminus of the vaccine peptide using an EAAAK linker in order to potentiate antigen-specific immune responses. The 50S ribosomal protein L7/L12 (RL7_MYCTU, UniProt ID: P9WHE3) was incorporated as an adjuvant at the N-terminal of the vaccine construct to enhance immunogenicity. The adjuvant RL7_MYCTU was an ideal adjuvant for enhancing cell-mediated immunity in this MEV vaccine construct as it is well-documented to have the ability to act as a potent immunostimulatory molecule inducing cytokine production, T cell activation and IFN-γ secretion (25). The final vaccine peptide generated consisted of 423 amino acid residues. The immunogenic peptide identified through epitope-mapping has been patented for further vaccine development.

### 3.2 Physiochemical properties and solubility prediction

The molecular weight (MW), theoretical isoelectric point (pI), and half-life of the final protein [as assessed in mammalian reticulocytes (*in vitro*) and in yeast and *E. coli* (*in vivo)*] is summarized in **Table 2**. The protein demonstrated good solubility upon expression, with a solubility score and an Abs 0.1% (1 g/L) value of 0.505. Furthermore, the instability index (II) was calculated as 36.67, classifying the protein as stable, as proteins with an II >40 are typically considered unstable.

### 3.3 Secondary-structure analysis and tertiary-structure modeling of the chimeric MEV construct using Swiss-model server

**Figure 2:**
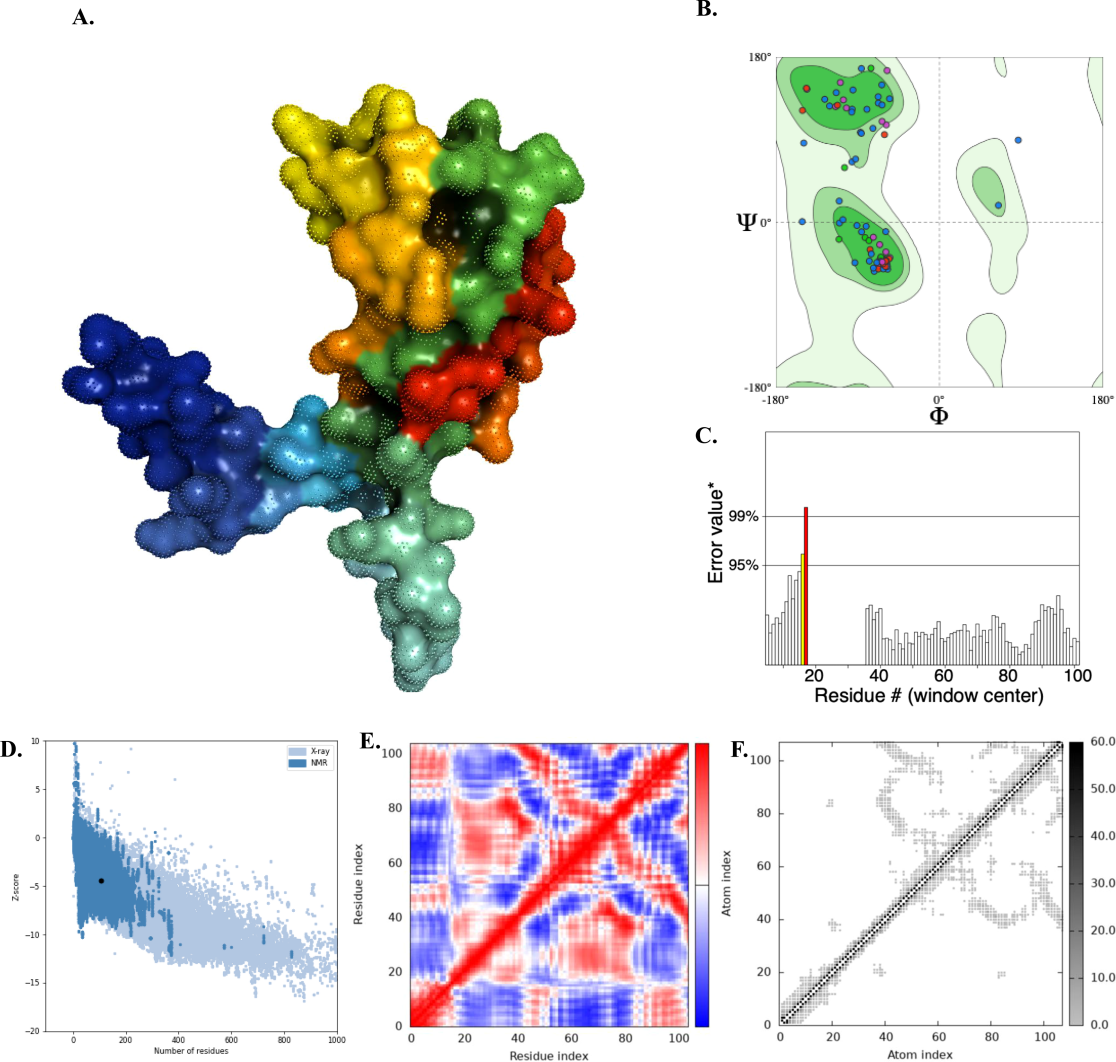
Protein 3D modelling, refinement and validation of designed MEV. **(A)** 3D structure of *LpKWTc001* multi-epitope-based vaccine design for MAC species. **(B)** Ramachandran plot **(C)** ERRAT analysis report. **(D)** Xray based on ProSA (Protein Structural Analysis). The Z-score (dark spot) value was **4.41**, within NMR (dark blue) and X-ray (light blue. **(E)** Covariance matrix indicating coupling between pairs of residues, i.e. whether they experience correlated (red), uncorrelated (white) or anti-correlated (blue) motions. **(F)** The elastic network model defining which pairs of atoms are connected by springs. Dots are coloured according to their stiffness, the darker greys indicate stiffer springs and vice versa.

The Swiss-Model server was used to generate the two tertiary structure models for the designed chimeric protein. Among these, the **MSVQ63Q02.1** model was identified as the best as it represented the full-length MEV construct and achieved a favorable MolProbity score (1.73) and low clashscore (3.64) and thus was selected for presentation (**Table 1**, **Figure 2A**). Ramachandran plot analysis indicated that 88.35% of the residues were in favoured regions, with only 1.94% in disallowed regions (**Table 1**, **Figure 2Bb**). The quality and accuracy of the refined 3D model were evaluated using ProSA-web and ERRAT. The ERRAT analysis reported an overall quality factor of 97.5% (**Figure 2C**), while ProSA-web yielded a Z-score of -4.41 (**Figure 2D**), confirming the reliability of the refined vaccine protein model. The internal dynamics of the MEV model were further examined using normal mode analysis (NMA) via the iMODS server. The covariance matrix (**Figure 2E**) illustrated patterns of correlated and anti-correlated motions between residues, while the elastic network model (**Figure 2F**) highlighted stiffness variations across residue connections, indicating regions of structural rigidity and flexibility.

### 3.4 IFN-γ inducing epitope prediction

**Figure 3:**
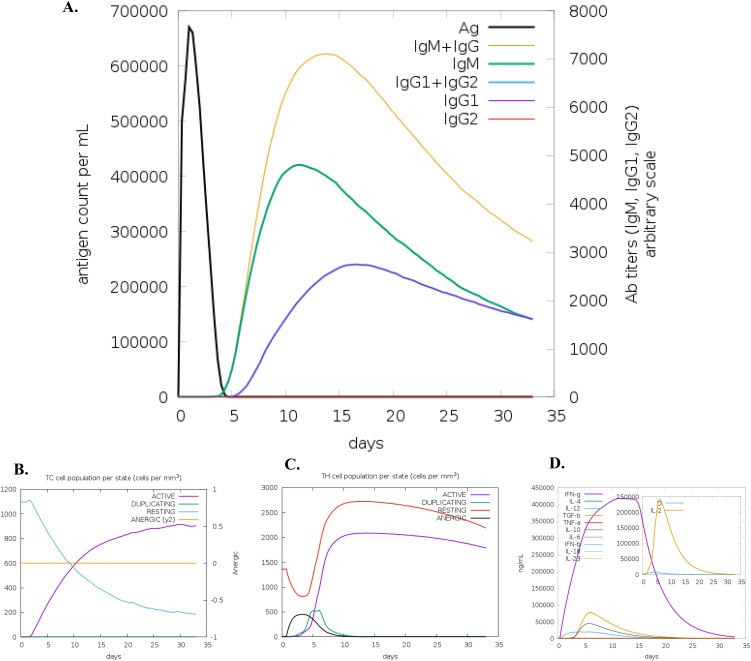
C-ImmSim simulation of the cytokine levels induced by the vaccine. **(A)** Antigen and immunoglobulins (antibodies are sub-divided per isotype); **(B)** CD8 T-cytotoxic lymphocytes count per entity-state; **(C)** T helper (TH) cell population, and **(D)** Concentration of cytokines and interleukins.

This prediction was consistent with the simulated level of IFN-γ produced after immunization with the peptide using the C-ImmSim server (http://150.146.2.1/C-IMMSIM/index.php). The MEV model we designed managed to elicit a significant increase in T cell population following immunization. Furthermore, the antibody levels (IgM+IgG, IgG1+IgG2, IgM, and IgG) were found to increase during immunizations, accompanied by a decrease in antigen count (**Figure 3A**). Additionally, both CTL and HTL populations increased following secondary and tertiary immunization (**Figures 3B–D**).

### 3.5 Codon optimization and the *in vitro* expression simulations

**Figure 4:**
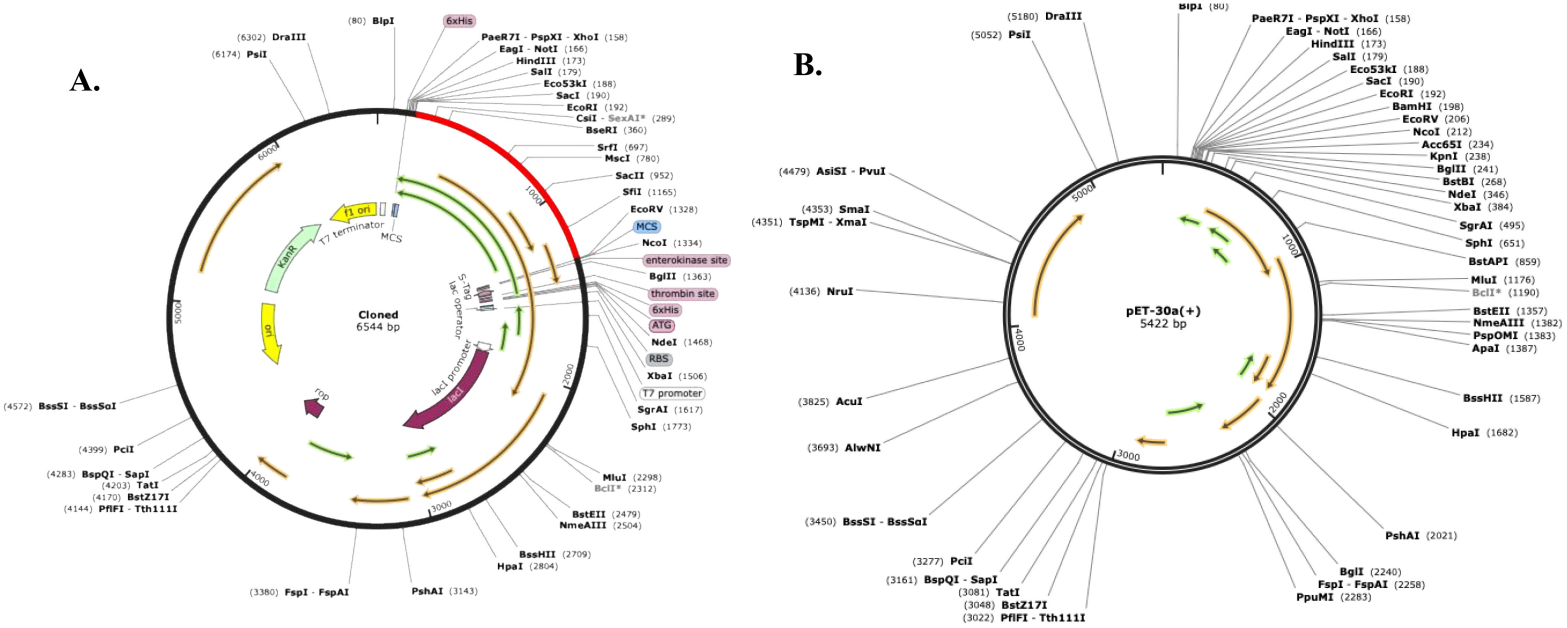
Codon optimisation and *in silico* restriction cloning of the multiple-epitope vaccine construct into the expression vector *pET30a(+)*. The codon sequence of each multi-epitope vaccine was inserted in the multiple cloning site (MCS) of the **(A)** *pET30a(+)* expression vector using the Snapgene sequence alignment tool; **(B)** Final clone with fragment *MEV-LpKwTC001*.

The Codon Optimization Tool (ExpOptimizer) tool (https://www.novoprolabs.com/tools/codon-optimization), was used for the multi-epitope vaccine model to enhance efficient translation and optimization of the codons for maximal expression in the prokaryotic host system *E. coli* (strain K12). In codon-optimized sequences of the designed vaccine *(MEV-LpKwTC001)*, the codon adaptation index values were 0.80, and the GC content value 64.14%. Additionally, the adapted codon sequences were optimized with sticky end restriction sites of *HindIII* and *NcoI* at the N-terminus and C-terminus to facilitate restriction and cloning and inserted into the recombinant plasmid vector, *pET-30a (+)*, using the Snapgene tool to design and effective cloning strategy (**Figure 4**).

## 4 Discussion

Paediatric administration of BCG vaccine is practiced in Zimbabwe. However, with *M. tuberculosis* being endemic and with the rise of NTM infections, there is a great need for new vaccines and booster vaccines for the BCG vaccines to fight both tuberculosis in adults as well as NTM infections particularly MAC infections. The rise of drug-resistant mycobacteria, limited BCG efficacy, and the need for vaccines targeting both humans and animals highlight significant challenges (33–35). The focus has recently shifted towards the development of subunit vaccines as they are associated with better safety profiles and are logistically more feasible, effective vaccines candidates that can be used to control MAC-related infections (36). Bioinformatics (*in silico*) is a good option to be used in designing and development of vaccines and diagnostics for newly emerged pathogens. The use of this approach reduces the time and cost. In order to construct a potent vaccine and effective diagnosis, understanding of the epitope and antibody interaction is required.

This work therefore focused on the *in-silico* design and development of a multi-epitope vaccine peptide generated using different MAC species (*MCOL, MAV, MINT*) and antigens (85A, 85B, and 85C) and has a potential for cross-protection (prophylactic and therapeutic). The proteins that we selected had exhibited potential to be vaccine candidates for *in vitro* studies (32). Epitope mapping of Ag85 protein complex has identified distinct peptides capable of stimulating human T cells, highlighting specific regions that could potentially trigger protective immune responses (37). More than 50% of vaccine candidates development for TB to date, some in advanced clinical trials, incorporated Ag85 (32). Conservation of the Ag85 complex across mycobacterial species also highlights their potential for cross-protection against related pathogens, including those in the MAC (30, 31).

We identified THL epitopes from selected proteins and fused them using linkers to create a multi-epitope peptide. Immuno-informatics showed the vaccine candidate contains many high-affinity MHC Class II epitopes from ag85B. Notably, multi-epitope vaccines are often poorly immunogenic and typically require adjuvants (38, 39); however, the designed protein demonstrated comparable antigenicity scores with or without an added adjuvant sequence. The antigenicity of the final sequence (including the adjuvant sequence) was shown to be probable antigen with a bacteria and also to be non-allergenic. we identified epitopes with accessibility, flexibility, hydrophilicity, and antigenic profiles for the Ag85. The vaccine candidate has a molecular weight of 41.5 kDa and is predicted to be soluble upon expression, aligning with its simulated immunogenicity. Solubility in an *E. coli* host is crucial for biochemical and functional studies.

Based on the predicted GRAVY score, which assesses maintenance ability in hydrophilic or hydrophobic environments, our MEV model displayed negative GRAVY value, suggesting a higher structural stability in a hydrophilic environment. This aspect can be correlated with solubility, critical in determining *in vitro* protein expression. Consequently, *MEV-LpKwTC001*, which demonstrated the highest solubility score, was selected for expression in *E. coli*. Furthermore, the predicting protein solubility is crucial for the selection of highly effective candidate proteins, as it can help avoid protein aggregation, which adversely affects biological activity and can lead to failures in the recombinant protein pipeline. The theoretical pI of 10.27 indicates the protein is alkaline, and the predicted instability index confirms its stability upon expression. The aliphatic index highlights the presence of hydrophobic aliphatic side chains, suggesting thermal stability, which is ideal for use in endemic regions like sub-Saharan Africa. These properties show that this is a potential vaccine design. To the best of our knowledge, no vaccine candidate is in phase III, nor licensed for use in the NTM infection with MAC species. To improve the immunogenicity of the vaccine antigen, we inserted adjuvant and linker sequences between the previously predicted epitopes to increase antigenicity. The fact that it has no allergenic properties further confirms its potential as a vaccine candidate.

Secondary structure analysis shows the protein is primarily composed of coils (67%), with 48% of residues disordered. These structural features, including natively unfolded regions and alpha-helical coiled coils, are known to serve as “*structural antigens*” capable of folding into native structures and being recognized by infection-induced antibodies. To evaluate conformational changes of the MEV, protein flexibility was examined using NMA. The MEV showed that a greater part of its peptide chains have high rigid regions which are crucial in the protein’s functional dynamics. The 3D structure, refined to improve its quality, exhibited favourable characteristics in the Ramachandran plot, with 85.16% of residues in allowed regions and minimal outliers, confirming the model’s reliability and suitability for vaccine design.

Immune simulation showed responses typical of a strong immune reaction, with increased activity after repeated antigen exposure. Following infection with MAC species, IgG1, IgG3, and IgE antibodies are critical for protection, and the vaccine candidate effectively stimulated memory B-cells and T-cells, with B-cell memory lasting several months. The simulations show that THL cells were strongly activated, and levels of IFN-γ and IL-2 spiked after the first injection, staying high with subsequent doses. This suggests strong TH cell activity and efficient antibody production, supporting a robust humoral response. The diversity of the immune response, indicated by the Simpson index, reflects the chimeric peptide’s design, which includes multiple B and T-cell epitopes. The dominant IFN-γ-driven TH1-type response, seen in naturally immune individuals, involves higher levels of TH1 cells, cytotoxic CD8+ T cells, neutrophils, and macrophages, further highlighting the vaccine’s potential effectiveness.

After obtaining the candidate vaccine, validating a candidate vaccine begins with screening for immunoreactivity using serological analysis, which requires expressing the recombinant protein in a suitable host. *E coli* expression systems, particularly strain K12, are preferred for producing recombinant proteins. To ensure high-level expression of the vaccine protein, codon optimization was performed *in silico*, yielding a favourable codon adaptability index (0.80) and GC content (64.14%).

While our vaccine candidate demonstrated favourable protein characteristics and strong immunogenicity, our study had certain limitations. Since the candidate was designed using human MHC epitopes, its efficacy must be assessed in a humanized mouse model. Additionally, we did not evaluate vaccine efficacy *in vitro*, which remains a limitation of our approach. However, this work represents a crucial foundational step toward experimental vaccine development. Notably, similar multi-epitope vaccines designed through utilization of immunoinformatic tools *in silico* have demonstrated strong immunogenicity in both *in vitro* and *in vivo* models, supporting the reliability of these methods (40–42). Moving forward, to advance the development of a preventive and therapeutic vaccine for MAC, we will validate the proposed vaccine through *in vivo* and *in vitro* studies to corroborate the predicted immunogenic potential.

Additionally, population coverage analysis for our vaccine candidate focused on HLA-DRB alleles. While this approach provides a robust estimation of coverage in African populations, the exclusion of HLA-DQ and HLA-DP loci may result in an underestimation of total MHC class II diversity, particularly in non-African populations. Future studies incorporating these additional loci are warranted to refine global population coverage estimates.

This study highlights a novel vaccine construct capable of eliciting a strong immune response against MAC species, potentially serving as a prototype for vaccines targeting other emerging infectious diseases. Vaccination is an important strategy to induce an immune response against the pathogen by specifically inducing the adaptive immune system. However, current challenges such as the absence of approved vaccines for MAC species, limited epitope-based research, and the lengthy development timelines and high costs associated with traditional vaccine approaches present significant gaps. Addressing these gaps is essential for comprehensive disease control and the reduction of MAC-associated morbidity across human and animal populations. This approach accelerates and lowers the cost of developing diagnostics and vaccines for MAC species, aiding future studies on epitope-based solutions to tackle the NTM challenge.

## Data Availability

The datasets presented in this study can be found in online repositories. The names of the repository/repositories and accession number(s) can be found below: https://www.ncbi.nlm.nih.gov/, BioProject ID PRJNA1205738.
